# Early Prediction of Asthma

**DOI:** 10.3390/jcm12165404

**Published:** 2023-08-20

**Authors:** Sergio de Jesus Romero-Tapia, José Raúl Becerril-Negrete, Jose A. Castro-Rodriguez, Blanca E. Del-Río-Navarro

**Affiliations:** 1 Health Sciences Academic Division (DACS), Juarez Autonomous University of Tabasco (UJAT), Villahermosa 86040, Mexico; 2 Department of Clinical Immunopathology, Universidad Autónoma del Estado de México, Toluca 50000, Mexico; dr.raulbecerril@gmail.com; 3 Department of Pediatric Pulmonology, School of Medicine, Pontificia Universidad Católica de Chile, Santiago 8330077, Chile; jacastro17@hotmail.com; 4 Hospital Infantil de México Federico Gómez, Mexico 06780, Mexico; blancadelrionavarro@gmail.com

**Keywords:** asthma, epigenetics, biomarkers, predictive models, machine learning

## Abstract

The clinical manifestations of asthma in children are highly variable, are associated with different molecular and cellular mechanisms, and are characterized by common symptoms that may diversify in frequency and intensity throughout life. It is a disease that generally begins in the first five years of life, and it is essential to promptly identify patients at high risk of developing asthma by using different prediction models. The aim of this review regarding the early prediction of asthma is to summarize predictive factors for the course of asthma, including lung function, allergic comorbidity, and relevant data from the patient’s medical history, among other factors. This review also highlights the epigenetic factors that are involved, such as DNA methylation and asthma risk, microRNA expression, and histone modification. The different tools that have been developed in recent years for use in asthma prediction, including machine learning approaches, are presented and compared. In this review, emphasis is placed on molecular mechanisms and biomarkers that can be used as predictors of asthma in children.

## 1. Introduction

The global prevalence of asthma has been increasing in recent years. Although it has stagnated, several publications in the last five years have suggested an overall prevalence of pediatric patients with asthma symptoms of approximately 10%, and a prevalence of 6–7% in adults. There are marked regional variations, with increases in many low- to middle-income countries. The risk factors associated with asthma prevalence include exposure to respiratory viruses, environmental pollutants, stress, obesity, genetics, gender, indoor allergens such as dust mites, and exposure to tobacco, among others [1].

It is a disease with significant variability, especially as the illness develops. Asthma spans a wide spectrum, with some patients experiencing remission of symptoms, whereas symptoms persist in others throughout their lives. More than 70% of patients with asthma present clinical manifestations during the first six years of life. It is worth mentioning that children who present with asthma at the age of 7 years have a 67–75% chance of becoming asymptomatic in adulthood [2].

Due to the contrasting nature of the disease, diagnosing asthma in children under the age of five is challenging, owing to the non-specificity of the clinical symptoms, as well as the lack of a comprehensive definition and a gold-standard diagnostic or prognostic marker [3]. Preschool-onset wheeze is highly prevalent, with different phenotypes and variable prognoses. An estimated 25–40% of all children wheeze in their fcirst seven years of life and may present six longitudinal patterns of wheezing in the first year of life, as described in association with asthma later in life [2,4].

The early identification of wheezing in children younger than five years old can provide valuable information to parents and medical professionals and aid in the early stratification and close monitoring of patients at risk of asthma [4]. Preschool is a crucial period for the development of the immune system and lung growth. The interrelation between genes and the environment has called attention to the vulnerability of the respiratory systems of infants to environmental exposure to various triggering factors [5].

The complexity of a diagnosis of asthma at preschool age leads to uncertainty in clinical decision making, potentially favoring the under- or overdiagnosis of asthma [6,7].

Prediction models for childhood asthma are helpful for identifying likely future asthma patients from high-risk groups; children in preschool who develop symptoms could benefit from early diagnosis and intervention [6].

In this review, we describe predictive factors, epigenetic phenomena in asthma, the use of biomarkers, prediction models, and machine learning.

## 2. Predictive Factors for Asthma

Different parameters have been used in asthma prediction models, the most important of which are age, gender, wheeze frequency, the presence of eczema, rhinitis, non-cold-related wheeze, peripheral blood eosinophilia, skin testing, specific IgE, biomarkers, gene expression, exercise-related cough/wheeze, and aeroallergen-related cough/wheeze [8].

### 2.1. Age

The development and trajectory of asthma are considered dynamic processes that comprise multiple phenotypes and affect patients of all ages. The appearance of signs and symptoms depends on the coursing age, with varying clinical expressions, leading to different diagnoses and necessitating different assessments and treatment strategies [9].

It has been stated that the age at which the first clinical manifestation of the disease appears is an important marker for the phenotypic characterization of asthma. In a cohort comprising five European databases, adult patients were diagnosed with asthma between 2008 and 2013 and categorized according to the age of asthma onset: childhood-onset age (<18 years old), adult-onset age (18–40 years old), or late-onset age (>40 years old). Compared to childhood-onset subjects, those with adult-onset asthma were reported to be at higher risk of overweight or obesity and at lower risk of atopic diseases, and patients with late-onset asthma were reported to have uncontrolled asthma more frequently [10].

According to a study of a population that was part of FinEsS (Finland–Estonia–Sweden), the median age for the diagnosis of allergic asthma was 19 years, and that for non-allergic asthma was 35 years. The incidence of allergic asthma was high in the 0–9 age group (1.8/1000/year) and lower in the 50–59 age group (0.6/1000/year). On the other hand, the incidence of non-allergic asthma was lower and remained so during childhood and young adulthood (0.7/1000/year), with a marked increase in the 50–59 age group (2.4/1000/year). This study reasserts the concept that the mechanisms involved in childhood-onset or early-adulthood asthma are distinct and function as separate entities from late-onset asthma [11].

### 2.2. Gender

Atopy and the male gender are described as risk factors for wheezing and asthma in childhood; after the preschool stage, this incidence decreases [12]. A review aimed at identifying possible gender differences in children and adolescents with asthma as determinants of the incidence and prevalence of asthma reported that boys have a higher prevalence of childhood wheeze and asthma than girls. In several studies, up to two out of three patients with childhood asthma or wheezing were male, and one out of three were female, with ORs of 1.4–1.6. In adolescents, the trend is different, with a higher prevalence in females than males among patients who start to show signs of asthma or wheezing in adolescence [13].

Dysanaptic lung development, a physiological discrepancy between lung parenchymal growth and airway caliber, is more prominent in boys than in girls [14,15]. In obese children and women with asthma, airway dysanapsis has been associated with increased symptomatology and decreased response to inhaled steroids [15]. As a dimensional difference in the respiratory system that is usually associated with gender, dysanapsis has clinical implications and may influence the pathogenesis of asthma and exercise tolerance [16].

Oscillations in sex hormones play an important role in women, increasing in severity and prevalence after reaching puberty. Estrogens and progesterone affect the immune response and pathogenesis of asthma, airway hyperresponsiveness, and type 2 inflammation involving eosinophils, ILC2, IL-13, and IL-5. They also act on the release of IgE in allergic inflammation and non-type 2 inflammation [17].

A novel asthma phenotype was proposed for females who menstruated before the age of 11 and grew up with obesity between the ages of 6 and 11 [18]. As described by the Tucson Children Respiratory Study (TCRS), these girls were more likely to develop new asthma symptoms at the age of 11–13 years old [19].

### 2.3. Hygiene Hypothesis

Since its initial description, the hygiene hypothesis has aroused interest in the scientific community and has been the subject of multiple investigations. According to observations from the original study in the United Kingdom, it is likely that a decrease in the number of family members, together with an increase in hygienic habits, reduced the chances of co-infection in young families, favoring an increase in the clinical expression of allergic disease [20]. Significant advances in epigenetics, a paradigm shift with new classes of effector and regulatory immune cells such as ILC2, and studies on biomarkers and different phenotypes allow us to understand the complexity of the immune response in the development of allergies and asthma [21].

Different studies have documented the association between early exposure to high bacterial loads (or the lipopolysaccharides they contain)—as occurs for those staying in daycare centers, those with a dog at home, and those living on farms—and a decrease in the development of allergic diseases and asthma. Various factors can modify this “farm effect” response, such as host genotype, age, presence of allergic sensitivity, and interactions [21,22].

The “old friends” hypothesis argues that microorganisms and macroorganisms such as parasitic helminths co-evolved with the human immune system, making this hypothesis similar to the “hygiene hypothesis”. The diversity in the composition of the gut microbiota, which contains approximately 22.2 million genes, is essential for homeostasis. Events that favor intestinal dysbiosis, such as the early use of antibiotics, infections, and changes in diet, cause alterations in the resident bacteria of the human intestines, modifying the normal microbiota through buffer mechanisms that promote allergic diseases. The “microflora” hypothesis is considered as another extension of the “hygiene hypothesis” [23]. The diversity and duration of microbial exposure in childhood are important for the induction of immunological tolerance; epidemiological studies have reported a correlation between early microbial exposure to allergens and decreased presentation of asthma and allergic rhinitis at a later age [24].

Changes in lifestyle in recent decades—mainly in industrialized countries—that favor modifications in the environment, microbiome, and regulation of the immune response have been described in the context of the so-called biodiversity hypothesis. Alterations in the immune response caused by microbial imbalance lead to a high risk of inflammatory diseases, including allergic diseases, such as asthma and allergic rhinitis [25]. In summary, the hygiene hypothesis continues to be valid and adaptable to innovations in scientific knowledge [21].

### 2.4. Allergic Comorbidity

The allergic phenotype associated with asthma exhibits a diverse range of characteristics that are influenced by a complex interplay of environmental, genetic, and psychosocial factors. The atopic march model describes the sequential manifestation of childhood allergic diseases, starting with atopic dermatitis (AD) and potentially progressing to asthma and allergic rhinitis, with the possibility of food allergies preceding respiratory allergies [26]. However, a study of two cohorts (UK cohort, *n* = 9801) showed a lower prevalence of this classical atopic march, e.g., eczema, wheeze, and rhinitis, as only a small fraction of symptomatic children seemed to follow this trajectory [27].

The Melbourne atopy cohort study (MACS) shed light on the increased risk of asthma at the age of 12 years for children sensitized to house dust mites, especially among those with early wheezing or eczema. Moreover, the study revealed a threefold-elevated risk of developing asthma and allergic rhinitis in later childhood and adolescence for children with persistent early-onset atopic dermatitis [28].

Further investigations, such as the Avon Longitudinal Study of Parents and Children (ALSPAC) and the Prevention and Incidence of Asthma and Mite Allergy (PIAMA) study, emphasized the association between persistent or later-resolving atopic dermatitis and an increased likelihood of coexisting asthma, high IgE levels, and familial history of atopy [29].

Familial atopy is consistently recognized as a significant predictor of asthma from childhood to adulthood, with the children of allergic parents manifesting rates of asthma that are two to three times higher. Between the ages of 4 and 20 years, among patients diagnosed with asthma, three-quarters were sensitized to at least one of five common aeroallergens [30].

The coexistence of allergic diseases, which is known as allergic multimorbidity, is prevalent, with asthma, allergic rhinitis, and eczema frequently overlapping. Multiple observational studies confirm this pattern, linking family history of allergy, childhood allergic symptoms, and cesarean section births as risk factors for allergic multimorbidity [31,32]. These allergic diseases share many genetic risk variants that dysregulate the expression of genes related to the immune response [33].

Allergic rhinitis, a common comorbidity in asthma, not only is a risk factor for asthma development, but also contributes to suboptimal asthma control [34]. In summary, these findings emphasize the intricate relationships among different allergic diseases and the need to consider genetic and environmental factors to comprehend their development and impact [31,32,34].

## 3. Epigenetics in Asthma

It is now widely acknowledged that diseases have a component of genetic susceptibility, and environmental factors may modify individuals’ susceptibility [35]. Thus, prolonged exposure to various environmental, dietary, and lifestyle factors contributes to the development or prevention of diseases in subjects with certain genetic characteristics, i.e., those that are “susceptible”. Epigenetics describes the complex influence of the environment on gene expression without changes in DNA sequences [36]. Multiple studies have shown how epigenetic pathways impact various disease features, from asthma in children to asthma in adults [37].

### 3.1. Epigenetic Mechanisms

DNA methylation, microRNA expression, and histone modifications (post-translational) are the most prevalent epigenetic pathways that have been studied, and they play a regulatory function in asthma gene expression and immunological response [38].

### 3.2. DNA Methylation

Among the various epigenetic mechanisms that have been described, the best characterized is CpG DNA methylation, which refers to the inclusion of a methyl group and cytosine in a CpG dinucleotide, resulting in the conversion of cytosine into 50-methylcytosine [39]. DNA methyltransferases (DNMT1, DNMT2, and DNMT3) catalyze the methylation [40], and the methylation pathway generally primes the inhibition of transcription, while, in contrast, hypomethylation leads to an upregulation of gene expression [41,42].

### 3.3. DNA Methylation and Asthma Risk

Studies have revealed that links between epigenetic patterns and pro-inflammatory pathways could possibly be used to identify early asthma predisposition. Nadeau K. et al. found a link between regulatory T cells (Tregs) and greater methylation of the FOXP3 gene in asthmatic children exposed to more ambient pollution [43], and a separate study found a link between the methylation of acyl-CoA synthetase long-chain family member 3 (ACSL3) in children with prenatal exposure to polycyclic aromatic hydrocarbons and asthma symptoms [44].

In a study on newborns, *SMAD3* methylation in asthmatic mothers was linked to a higher risk of the newborns developing asthma in childhood [45]. A separate study demonstrated changes in the blood methylomes of children of asthmatic mothers; this could act as a risk factor for asthma development [46].

Cigarette smoke exposure modulates DNA methylation, as shown by the differing levels of methylation in fetal lung tissue and the placenta when the mother smokes or is exposed to smoke [47]; however, the exact mechanism by which these differentially methylated sites are linked to asthma is unclear.

### 3.4. MicroRNA Expression

MicroRNAs (miRNAs) are non-coding RNAs that typically consist of 18–25 nucleotides and influence up to 60% of mRNA translation through mRNA destabilization [48]. Due to the fact that miRNAs are implicated in post-transcriptional changes, their functions often depend on the association of miRNAs with their target genes, the site at which the miRNA is expressed inside the cell, the cell bloodline, the number of miRNAs, and the complementarity of the miRNA and the gene [49]; as regulators of the immune response, they play an essential role in the pathological evolution and development of asthma [50].

Wang et al. showed that *miRNA-451a* downregulation increases the expression of ETS1 in CD4+ T cells and increases the production of Th2 cytokines (IL-5 and IL-13), which may contribute to the differentiation of Th2 cells in children with asthma [51]. Likewise, dysregulation of the *miRNA-451a*–CDH11 axis is involved in pathological changes in bronchial tissue [52].

Another review demonstrated that abnormal expression of miRNAs (*miR-146a* and *miR-106b*) was related to the production of Th2 cytokines (IL-5 and IL-13), which are both involved in the pathogenesis of asthma in children [53]. In addition, *miR-15a* was linked to smoke exposure during lung development, and it regulated the expression of asthma-related genes, thus providing evidence of the probable fetal origin of asthma [54,55].

It is known that exposure to air contaminants has been associated with respiratory immune responses, which cause alterations in miRNA expression. In addition, chronic exposure to ozone leads to airway inflammation, which represents a potential risk factor for asthma development [56]. Fry et al. found that the inhalation of O_3_ was linked to the overexpression of several microRNAs in the bronchial airways, with a possible link to the inflammatory pathway in asthma [57].

Of these, *miR-21* is the most-well evaluated miRNA in asthma, and it is known that it can lead to Th1/Th2 imbalances through the downregulation of interleukin-12 (IL-12) and the overexpression of IL-4 [58]. Additionally, Th2 inflammation has been linked to the let-7 family of miRNAs [59], and the response of eosinophilia in asthma has been linked with two additional miRNAs (*miR-155* and *miR-221*) [60,61]. Therefore, it is evident that miRNAs play an important role in the pathogenesis and development of asthma.

### 3.5. Post-Translational Histone Modifications

Core histones wrap DNA to create a structured chromatin structure. Post-translational histone modifications such as acetylation, methylation, phosphorylation, ubiquitination, SUMOylation, and ADP-ribosylation constitute another epigenetic mechanism that is crucial in different illnesses, including childhood asthma [57].

Histone acetyltransferase (HAT) leads to histone acetylation, causing the chromatin structure to become loose and open to transcription factors, thus inducing the expression of genes; conversely, the activity of the enzyme histone deacetylase (HDAC) leads to gene silencing through histone deacetylation [58].

As an example, the acetylation, dimethylation, and trimethylation of histone H3 lysine 9 in the iNOS promoter can be implicated in its response, as shown in primary human vascular endothelial cells [42,62]. A separate study suggested that environmental particles that increase cellular acetylation may sustain or strengthen recall reactions to Th2 phenotypes that are implicated in several diseases, such as asthma and allergies [63].

In addition, in children with allergic asthma, the ratio of histone deacetylase and histone acetyltransferase (HDAC/HAT ratio) was found to be altered, and a higher severity of bronchial hyperresponsiveness was correlated with cellular acetylation activity [64]. Likewise, histone acetylation has been linked to the differentiation and function of Th cells. Enhanced histone acetylation in genes that encode Th2 cytokines in response to Th2 differentiation was observed, which led to increased Th2 cytokine production [65]. On the other hand, disease-specific enhancers that were selectively methylated in primary T cells isolated from peripheral blood—causing changes in healthy patients and asthmatics during Th2 differentiation—were prone to the development of dimethylation at histone H3 lysine 4 [66].

## 4. Biomarkers

### 4.1. Lung Function

According to a number of cohort studies, asthma is linked to abnormal lung function before the first wheezing episode and in the first several weeks of life [67]. Additionally, asthma remission and persistence can be predicted based on lung function [68]. However, although the standardized measurement of lung function is a common component of evaluations of school-aged children with asthma, it is rarely used in preschoolers due to its complexity.

The first study to address the link between lung function and risk of wheezing was the Tucson Children’s Respiratory Study (TCRS). Using the TCRS, Martinez et al. showed that diminished lung function as a result of a reduction in the caliber of the airway and changes in lung tissue possibly increased the risk of infant wheezing [67,69].

The Copenhagen Prospective Studies on Asthma in Childhood (COPSAC) showed that decreased lung function at neonatal age (FEF50) is a risk factor for asthma at 7 years of age [70]. In addition, the Childhood Asthma Management Program (CAMP) study showed that, during childhood, a 10% higher FEV1/FVC ratio was associated with asthma remission during early adulthood (OR 4.62); on the other hand, only 1.4% of children with FEV1/FVC ratios less than 70% would experience asthma remission, making this ratio highly predictive of asthma persistence [71].

The Perth cohort also showed a link between diminished lung function and a consequent diagnosis of asthma; the researchers discovered that infants with restricted airways had a higher risk of developing asthma by the age of two [72]. On the other hand, the lung function z-score was considerably worse in children with persistent asthma in comparison with non-asthmatic children at age 11 [73]. Australian and Norwegian cohorts demonstrated diminished lung function at as young as 1 month in children who developed persistent wheezing later in childhood [74]. Recently, a small Chilean cohort study showed that, at preschool age, alterations in some impulse oscillometric parameters (AX, R5–R20 difference, and R5) could predict abnormal spirometry and BDR at school age [75].

### 4.2. Bronchoscopy

Although the evaluation of bronchoalveolar lavage (BAL) fluid and/or bronchial biopsies has been used as the gold standard for evaluating airway inflammation, these biopsies are impractical due to their invasiveness.

Adult asthma is characterized by eosinophilic inflammation and thickening of the reticular basement membrane (RBM) [76,77]. Endobronchial biopsy studies showed that, at 2–3 years old, children with severe recurrent wheezing already had these characteristics [78], but they seemed to be absent in wheezing infants at the age of 12 months [79], suggesting that pathological changes in the airway could start earlier than 3 years of age.

A systematic review of 39 studies (involving 2390 children under 18 years old) demonstrated that, at a mean age of 12 months, eosinophilic/neutrophilic airway inflammation and remodeling were absent in wheezers; however, remodeling (RBM thickness and increased area of smooth muscle in the airway) and airway eosinophilia were documented in older preschoolers (mean: 2.5 years). Children of school age experienced this with greater severity. Preschool wheezers with and without atopic dermatitis had similar RBM thicknesses. Seven studies found a correlation between airway remodeling and lung function, another three found a correlation with FeNO, and one found a correlation with HRCT scans. In addition, in patients without remodeling, eosinophilic inflammation was not observed [80].

In a recent study, using fiber optic bronchoscopy, Fayon et al. evaluated markers of bronchial remodeling (epithelial integrity, thickness of the reticular basement membrane (RBM), mucus glands, fibrosis, bronchial smooth muscle area (BSM), density of blood vessels, and RBM–BSM distance) in severe preschool wheezers to predict the exacerbation of wheezing after a year of biopsy. They found a two-class model using latent class analysis; the class with increased smooth muscle, blood vessel density, and RBM thickness and decreased mucus glands, fibrosis, and RBM–BSM distance was linked to significantly more uncontrolled asthma symptoms and a shorter time until the first exacerbation within the following 12 months. Therefore, the evaluation of bronchial remodeling through bronchoscopy may be used to identify severe preschool wheezers at risk of subsequent exacerbations [81].

It is known that reticular basement membrane thickening can be present in allergic bronchial asthma and in other bronchial illnesses, such as primary ciliary dyskinesia and cystic fibrosis. In a study of multiple regression models, Koucký et al. showed that changes in reticular basement membrane morphology resulted in diminished lung function by evaluating the z-scores of FEV1 and the FEV1/FVC ratio [82].

Therefore, the evaluation of bronchial remodeling through bronchoscopy could be used to predict the subsequent development of lung function in terms of subsequent exacerbations of wheezing in children; however, the invasive nature of such studies is a limitation for their application, as opposed to noninvasive biomarkers that can reflect remodeling.

### 4.3. Fraction of Exhaled Nitric Oxide (FENO)

Nitric oxide is produced in the airway by epithelial cells as a result of the upregulation of nitric oxide synthase induced by IL-13 during allergic inflammation [83,84]. FeNO is a noninvasive marker, which is reproducible, easily measurable, and one of the most studied biomarkers of eosinophilic airway inflammation [85,86].

Several studies have demonstrated that preschoolers with wheezing and probable asthma have greater levels of FeNO than those with no symptoms or possibly temporary wheezing symptoms [87,88]. In a longitudinal study, Elliot et al. showed that FeNO concentrations of ≥30 ppb at preschool age were associated with poor lung function and could predict the continuation of wheezing symptoms at three years (specificity of 94% and sensitivity of 77%) [89]. In another study among children between 3 months and 4 years old, it was shown that, in children with asthma at school age, the levels of FeNO were elevated in comparison with those in children without asthma; for every increase of 5 ppb in FeNO, the odds ratio (95% CI) for asthma increased by 2.44. To evaluate the predictive value of FeNO, the API was modified by adding FeNO levels instead of the blood eosinophil count; when this modification was used, the children who were at risk had a 58.0% probability of asthma development, whereas the negative predictive value was 78.2%, which was comparable to the classical API [90].

Two studies using online FeNO showed a significantly higher level in infants and preschoolers with the positive classical API. In Spanish infants (mean age: 12 months), those with a positive API had higher levels than those with a negative API (median (IQR) of 12.3 (14.8) ppb vs. 4.1 (7.9) ppb, respectively; *p* = 0.016) [91]. In Argentinian children (5–36 months old), the numbers were a median (range) of 13.5 ppb (0.7–31) vs. 5.6 ppb (0.1–20.8), respectively (*p* < 0.01) [92].

In the PIAMA study, the authors showed that, at the age of 4, FeNO and IgE could possibly predict asthma development at 8 years, regardless of medical history [93]. In a different study with the same PIAMA cohort, FeNO was increased in children who experienced persistent wheezing in comparison with those with transient wheezing phenotypes, but the elevation was only present in children with atopic sensitization [94].

Therefore, FeNO is an important biomarker in the early prediction of asthma with eosinophilic inflammation; however, it might not be sufficient for investigating the wide spectrum of preschool wheezers.

### 4.4. Allergy Assessment (Total and Specific Immunoglobulin E)

IgE is an antibody synthesized by plasma cells in response to an antigenic stimulus; it induces type 1 hypersensitivity reactions and plays a critical role in the pathogenesis of allergic asthma. In mast cells and basophils, it binds to IgE receptors to produce cytokines that mediate T2 responses, which are characteristic of allergic asthma [95]. Based on this, the role of IgE and the early sensitization of its relationship with asthma have been extensively studied.

A recent longitudinal review demonstrated that IgE levels of ≥0.24 kU/L in cord blood taken at birth were significantly associated with a 2.6-fold increase in the risk of asthma, elevated FeNO levels, and allergic sensitization. Nevertheless, the IgE levels in cord blood were not significantly correlated with measures of pulmonary function, suggesting that IgE from cord blood could be used to identify neonates who might develop allergic asthma later on [96]. Another study that used two independent birth cohorts—those of the Melbourne Atopic Cohort Study (MACS) (*n* = 620) and the population-based LISAplus from Germany (*n* = 3094)—evaluated the relationship between allergic airway illness and sensitivity to food, aeroallergens, or both. As a result, in both groups, food sensitization (with or without aeroallergen sensitization) was associated with a strong prediction of asthma during the first two years of life [97]. In a multivariate analysis, Boersma et al. proved that sensitization to inhalant allergens had a predictive value of 86% for asthma [98]. Similarly, using the Childhood Origins of ASThma (COAST) study from the USA, Anderson et al. demonstrated that peripheral blood eosinophilia and early-life aeroallergen sensitization were both strong predictors of asthma inception [99].

Additionally, early exposure to environmental antigens in the first few years of life has been described as an important factor in sensitization. Some studies have described early exposure to pets as a protective factor against the development of asthma and allergic diseases, but other studies have linked it to a worsening of symptoms. In the Danish National Birth Cohort, exposure to dogs was associated with a marginally lower risk of atopic dermatitis and asthma, leading to the conclusion that the relationship between early exposure to animals and allergic diseases can be modified by the presence of factors such as a history of asthma or allergy, time, and source of exposure [100]. In the Avon Longitudinal Study of Parents and Children (ALSPAC) (*n* = 4706), at the age of 7 years, a 6% reduction in wheezing in children with early exposure to cats was observed (OR = 0.94 (0.89–0.99)) [101].

In order to investigate the composition of the microbiome of fungi and bacteria in house dust, which could contribute to the presence of wheezing in childhood and allergenic sensitization, the LISAplus birth cohort study was carried out. In this prospective study, high exposure to a variety of fungi and bacteria in house dust was found to be inversely related to allergenic sensitization in patients at 6 years of age, as well as in those who continued wheezing at the age of 10 years [102].

It has been documented that microbial exposure in utero and in early childhood is essential for regulating the immune system’s response to environmental elements such as allergens and viruses.

As part of the PASTURE birth cohort study, sequenced samples of house dust were collected from patients’ rooms at 2 months of age. When they were subsequently evaluated at 10.5 years of age, an abundance of twelve genera of bacteria associated with a lower risk of asthma (*p* < 0.10) was found in the samples obtained. Confirming the data that were obtained, patients with greater protection against asthma at 10.5 years of age had greater phylogenetic diversity in the microbiota and bacteria according to the samples obtained from their homes [103].

Atopy is clearly a risk factor for the development of asthma, but an IgE or allergic sensitization test alone is insufficient to determine the risk of asthma.

### 4.5. Blood Eosinophils

Eosinophils are granulated cells that have the capacity to secrete a variety of inflammatory mediators and are implicated in the pathogenesis of many inflammatory disorders, including asthma [104,105]. Due to the low cost and wide availability of tests, blood eosinophils are considered a suitable biomarker [106], primarily because they predict asthma remission more accurately than specific IgE or skin-prick tests, and their absence was shown to be able to correctly predict more than 91% of remissions [107].

Despite studies linking blood eosinophilia with asthma [108], this method lacks sensitivity, making eosinophils a poor choice as a biomarker for the early prediction of asthma [109], but many studies have demonstrated their utility in phenotyping asthma, in the prognosis of exacerbations, and in the response to treatment with steroids and anti-eosinophilic monoclonal antibodies [110,111].

Another marker of eosinophil activity is eosinophil cationic protein (ECP), a protein with ribonuclease activity that can be measured in the serum and sputum. In patients with asthma, the levels of ECP are correlated with airway inflammation [106]. However, these levels may be higher in other atopic and nonatopic disorders, such as allergic rhinitis and viral respiratory infections, which are not particular to asthma [106,112].

### 4.6. Serum Periostin

Periostin is an extracellular matrix protein secreted by airway epithelial cells in response to IL-13, the Th2 inflammatory cytokines IL-4 and IL-13, and TGFβ. It may cause subepithelial fibrosis in asthma and has been associated with T2-high eosinophilic asthma [113]. Recent studies demonstrated that patients with eosinophilic asthma had higher serum periostin levels in the serum than those with non-eosinophilic asthma [114,115]. By analyzing data from the Swedish Global Allergy and Asthma European Network study, James et al. found that lower pulmonary function was associated with high levels of serum periostin in asthmatic patients [116]. It is known that, in children, the levels of periostin are two- to threefold higher than those in adults because periostin plays key roles in bone growth. However, according to a prospective cohort study, at the age of two years, periostin levels of ≥150 ng/mL could predict asthma at the age of six years [99]. A pilot study carried out among Chilean preschoolers showed that serum periostin levels were not significantly different between wheezing preschoolers with positive and negative classical API [117].

From these results, we can conclude that serum periostin is increased in patients with asthma. It is higher in atopic patients than in nonatopic patients and can be considered a diagnostic biomarker of bronchial asthma, but the exact role of periostin as an asthma predictor remains controversial.

### 4.7. Sputum Eosinophils

Eosinophils have been linked to allergic illness for as long as asthma has been scientifically researched, and their role in asthmatic inflammation is well known as a mediator of airway remodeling in asthma development [118,119].

Sputum eosinophilia has been extensively studied in the phenotyping of asthma and as a prognostic marker in asthmatic patients who have never used steroids; corticosteroid treatment usually results in a significant decrease in eosinophil levels in the sputum.

Although there have been some studies that have related blood eosinophils to wheezing in children, there is a lack of evidence that they are a suitable biomarker for the early development of asthma. In addition, although it has been shown to be safe, the collection of induced sputum in children is not easy, and the evaluation of the samples requires specific training [120].

### 4.8. Sputum Neutrophils

Another noninvasive method for assessing airway inflammation is sputum induction. It has been proven to be safe, but it is challenging to collect data from children using this method because it requires specific training and equipment, as well as patient coaching and cooperation [106,118,119].

One potential biomarker for predicting non-T2 asthma is the presence of neutrophils in the sputum, and many studies have linked the presence of high levels of neutrophils in the sputum with the severity of asthma, a relative lack of response to corticosteroid therapy, chronic exacerbations, and severe chronic airflow obstruction; however, this marker is not useful as a biomarker for the prediction of asthma.

### 4.9. Nitrites in Sputum

In comparison with FeNO, measuring nitrites in sputum (measured by using the Griess assay) is a cheap and simple method of measuring nitric oxide metabolites. Recabarren et al. showed, for the first time, that nitrites in sputum were significantly higher in patients with persistent asthma than in healthy schoolchildren (16.30 ± 8.6 vs. 10.25 ± 4.68 nmol/mL, respectively; *p* = 0.001). Moreover, the nitrite level in the sputum of children with severe persistent asthma was higher than that in those with moderate and mild asthma (32.83 ± 9.48 vs. 18.10 ± 1.96 vs. 11.84 ± 4.73 nmol/mL, respectively; *p* < 0.01 for the linear trend) [120]. The same research team found that, after three months of inhaled corticosteroid therapy, the nitrite levels detected in induced sputum decreased and were correlated with an improvement in the symptoms of clinical asthma. Between enrollment in and completion of the study, the sputum nitrite levels significantly changed (34.4 nmol/mL (IQR: 18.2–58.4) and 11.2 nmol/mL (6–20.1), respectively; *p* < 0.0001). Additionally, a significant correlation between the drop in sputum nitrite levels and improvements in clinical parameters (acute exacerbations (r = 0.361, *p* = 0.005), salbutamol use (r = 0.322, *p* = 0.013), emergency visits (r = 0.275, *p* = 0.033), and school absence (r = 0.41, *p* = 0.001)) from the time of enrollment to the end of the study was reported. However, there were no correlations between sputum nitrite levels and the results of a bronchial exercise test, peripheral blood eosinophils, or serum IgE levels. Therefore, measuring nitrite in induced sputum (a simple, inexpensive, noninvasive approach) may be a useful substitute for tracking the effectiveness of asthma medication in schools [121]. However, larger studies involving multiple populations must be conducted first.

### 4.10. Volatile Organic Compounds (VOCs) in Exhaled Breath Analysis

The analysis of exhaled breath condensate (EBC), which was introduced more than 20 years ago, is a noninvasive technique for obtaining samples of the airway. It examines volatile and nonvolatile compounds [122]. Volatile organic compounds are carbon-based chemicals that are easily converted into vapors or gases; they are present during physiological or pathophysiological processes and include nitric oxide products, hydrogen peroxide, leukotrienes, and cytokines [123].

Using chromatography, Caldera et al. characterized volatile organic compounds in patients with asthmatic and control children, obtaining compounds linked to oxidative stress, such as alkanes and aldehydes, in asthmatic children. These positive results showed that VOC profiles could successfully distinguish children with asthma from healthy children [124]. In the ADEM (Asthma Detection and Monitoring) study, 258 children were evaluated, and it was found that VOCs could be easily and safely obtained in exhaled breath analyses in preschool children; they demonstrated that VOC profiles could distinguish between healthy controls and preschool wheezers [125]. Another study demonstrated that exhaled breath analysis using a limited number of compounds (17 VOCs) was able to distinguish between preschool asthma and transient wheezers with an accuracy of 80% (sensitivity of 73% and specificity of 87%) after external validation [126].

These early results are encouraging and highlight the potential of using exhaled VOCs for the early diagnosis of asthma, but more studies are needed to evaluate their accuracy.

## 5. Prediction Models for Childhood Asthma

Over the last two decades, several asthma prediction tools have been developed to assess the risk of developing the disease in the future. These instruments have some elements in common, such as the frequency of wheezing episodes, eczema, parental history of asthma or allergy, and evidence of atopy (according to a skin prick test or specific IgE). Despite these similarities, the scores have shown variable performance in predicting asthma development [127].

We summarized eight of the most widely used tools for predicting asthma: the Asthma Predictive Index (API) from the Isle of Wight, PIAMA (Prevention and Incidence of Asthma and Mite Allergy), modified API (mAPI), ucAPI (University of Cincinnati API), the Asthma Prediction Tool (APT) from Leicester, Asthma Detection and Monitoring (ademAPI), and the Pediatric Asthma Risk Score (PARS) [8]. A summary of the characteristics of the predictive models is provided in Table 1.

It is important to remember that a diagnostic test’s clinical effectiveness is determined based on how it allows physicians to calculate the probability of a disease for specific patients by directly relating pre-test and post-test probabilities [128,129]. Hence, the calculation of the likelihood ratio (LR) is an effective tool that reflects the degree to which the pre-test probability increases or decreases [2]. In order to reflect the diagnostic precision of any prognostic model, the European Academy of Allergy and Clinical Immunology (EAACI) advises utilizing an LR interpretation of IgE sensitization tests. A similar strategy may also be utilized for predictive tools for asthma [130].

The first asthma prediction model was the original asthma predictive index (API), which originated from the TCRS in 2000, and two sets of prediction guidelines were established: a loose API and a strict API [131]. This is the best-regarded model and is used as a benchmark when comparing new models. According to reports, children with a positive severity index were seven times more likely to have active asthma when they reached school age (sensitivity, 28%; specificity, 96%; LR+, 7.6). The API has been suggested in childhood asthma guidelines [132,133,134], and it is one of the few tools that have been validated in new populations and in different cohorts [135].

In a birth cohort (*n* = 339) study that was recently carried out in Chile, the API was determined at 30 months of age, and its association with primary care physicians’ independent diagnoses of asthma was examined. After a multivariate analysis, the API+ was associated with an almost sixfold increase in the odds of an asthma diagnosis (adjusted OR = 5.7, 95% CI (2.6–12.3(). The API sensitivity was 48% and the specificity was 92%, with 61% PPV, 88% NPV, 6.4 LR+, 0.56 LR−, and 0.84 diagnostic accuracy. The adjusted odds for asthma were 11.4. This study suggested for the first time that the API could be used as a diagnostic tool and not only as a prognostic tool in toddlers and preschoolers [136].

Recurrent airway infections in children were some of the factors that the Isle of Wight [137] and PIAMA [138] studies included among their criteria, but these criteria can be confusing when it comes to wheezing. The PIAMA index is more difficult to calculate because several criteria are weighted differently, including socioeconomic data and parental education, and this information may be different according to the population in which this is applied [8,138].

Instead of using objective criteria for the clinical identification of allergic rhinitis, the original API was modified. The modified API [139] was evaluated in the high-risk COAST (Childhood Origins of ASThma) study, and a positive mAPI was shown to have a high predictive value after a positive test (LR+ 4.9 to 55) for asthma development at the ages of 6, 8, and 11 years. Similarly, the ucAPI [140] was developed in a high-risk atopic birth cohort, and it was used with other objective criteria (change in FEV1 of ≥12% post-bronchodilation or a methacholine challenge test with PC20 ≤ 4 mg/mL).

The APT [141] model, which was based on the Leicestershire cohort, used 10 predictive variables in preschool children who attended medical consultations with respiratory symptoms of ≥1 wheezing or chronic cough (cough without a cold or night coughing) in the last 12 months. This tool classified children into three groups according to their scores: scores ≤ 5 (low risk), scores in the range of 6–9 (medium risk), and scores ≥ 10 (high risk). These groups were shown to have 16%, 48%, and 79% risks of developing asthma later, respectively.

The ademAPI model added several parameters to the original API and replaced eosinophilia with specific IgE [8,142]. This model increased the positive LR from 7.4 (original API) to 8.8 (ademAPI), but due to the highly expensive and sophisticated predictors involved, it is very unlikely that this tool can be used in a generalized context [2].

The most recent model created was the Pediatric Asthma Risk Score (PARS), which used data from the Cincinnati Childhood Allergy and Air Pollution Study (CCAAPS, *n* = 762) and was replicated in the Isle of Wight birth cohort. This model uses six parameters, which include parental asthma, eczema, early wheezing, healthy wheezing, ancestry (black/African), and allergies, and it classifies children into low-risk (score: 0–4), medium-risk (score: 5–8), and high-risk (score: 9–14) groups [143]. However, the positive LR is low (3.25).

The prediction models for childhood asthma differ significantly in terms of the study design, study size, target population, predictor variables, school-age asthma diagnosis criteria, and statistical methods. The majority of prediction models that have been created have shown modest generalizability and moderate predictive performance when externally validated. Currently, only the original API, PIAMA, APT, and PARS indexes have been validated.

**Table 1:** Summary of asthma prediction models [8].

	Original API (Stringent Index) [131]	Isle of Wight [137]	PIAMA [138]	mAPI [139]	ucAPI [140]	APT [141]	ademAPI [142]	PARS (In IOW) [143]
Year of publication	2000	2003	2009	2013	2014	2014	2015	2018
Country	US	UK	Netherlands	US	US	UK	Netherlands	US
Number of children surveyed	1246	1034	2171	289	589	1998	202	589
Source population	General	High-risk	High-risk	High-risk	High-risk	High-risk	General	High-risk
Age (y) of asthma prediction	6, 8, 11, 13	10	7–8	6, 8, 11	7	6–8	6	7
Methods of building	Clinical index	Cumulate risk score	Logistic regression	Clinical index	Clinical index	LASSO regression	Logistic regression	Logistic regression
Number of predictors used	5	4	8	5	5	10	8	6
Sensitivity (%)	28 (at 6 years)	53	60	19 (at 8 years)	44	72	88	67
Specificity (%)	96 (at 6 years)	85	76	100 (at 8 years)	94	71	90	79
PPV (%)	48 (at 6 years)	68	23	87 (at 8 years)	60	49	90	36
NPV (%)	92 (at 6 years)	74	94	9 (at 8 years)	89	86	89	93
LR+	7.6	3.41	2.5	55	7.5	2.5	8.8	3.25
LR−	0.75	0.56	0.53	0.83	0.6	0.4	0.13	0.41
PREDICTORS								
Age					✓			✓
Gender			✓		✓			
Wheezing frequency	✓		✓	✓	✓	✓	✓	
Parental history of asthma or allergy	✓	✓		✓	✓	✓	✓	✓
Eczema	✓		✓	✓	✓	✓	✓	✓
Rhinitis	✓	✓			✓		✓	
Wheezing without colds	✓		✓	✓	✓	✓	✓	✓
Blood eosinophilia	✓			✓				
Skin-prick test		✓		✓	✓			✓
Specific IgE							✓	
Chest infections		✓	✓					
Parental medication inhalation			✓					
Parental education			✓					
Post-term delivery			✓					
Activity disturbance						✓		
Shortness of breath						✓		
Exercise-related wheeze/cough						✓		
Aeroallergen-related wheeze/cough						✓		
EBC biomarkers							✓	
VOCs							✓	
Gene expression							✓	
Ancestry								✓

API—asthma prediction index; APT—asthma prediction tool; PIAMA—Prevention and Incidence of Asthma and Mite Allergy; mAPI—modified API; ucAPI—University of Cincinnati API; ademAPI—Asthma Detection and Monitoring API; PARS—Pediatric Asthma Risk Score; IOW—Isle of Wight Cohort; PPV—positive predictive value; NPV—negative predictive value; LR+—positive likelihood ratio; LR−—negative likelihood ratio; EBC—exhaled breath condensate; Ig—immunoglobulin; VOCs—exhaled volatile organic compounds. Checkmark—✓.

## 6. Machine Learning

Machine learning (ML) is a branch of data science and artificial intelligence in which large amounts of data are processed to learn mathematical interactions [144,145]. Machine learning has been successfully applied in the medical field, mainly in the diagnosis and prediction of diseases [146,147]. In comparison with other traditional methods, ML has the ability to analyze massive amounts of data and create predictions with higher accuracy; it collects, evaluates, and processes data to discover trends and patterns and, potentially, determine new risk factors [148,149].

There are different types of algorithms used in the prediction of asthma in children, with the most relevant being the support vector machine (SVM), multilayer perceptron neural network, logistic regression, k-nearest neighbor, decision tree (DT), multilayer perceptron, random forest (RF), artificial neural network (ANN), and gradient-boosting machine [149,150]. These algorithms can be classified into two different classes: supervised and unsupervised learning [145,151].

Particularly in the last ten years, there has been an increase in the application of ML methods for asthma evaluation [152]; in addition, artificial intelligence may have advantages over traditional tools in terms of processing vast amounts of data and considering interactions among data, thus allowing for greater accuracy [153,154]. However, few studies have used ML to predict childhood asthma, with a greater number of studies focusing on adult asthma [155].

Using the PROBAST (Prediction model Risk of Bias Assessment Tool) checklist, Patel, D. et al. evaluated ten studies related to machine learning and asthma prediction. The values of the predicted performance metrics from ML models were greater than those obtained with traditional models in terms of sensitivity and specificity [150], but a substantial risk of bias was also demonstrated because of the heterogeneity of the data.

Another study directly compared the performance of conditional inference using tree-based machine learning with a current regression-based asthma prediction model, PARS, while using the same predictors. Using decision-tree-based conditional inference, ML had a higher predictive accuracy (AUC: 0.85; 95% CI: 0.81, 0.88; sensitivity = 47%; specificity = 93%) than that of the pediatric asthma risk score. However, the study has not been externally validated [156]. Likewise, Ekpo et al. assessed a total of 32 machine learning algorithms for asthma prediction in children; the analysis showed that ML performed better than the conventional asthma prediction tools, but due to the heterogeneity of the results and the different methodologies used, more research is needed [149].

Although artificial intelligence has been shown to be a viable option in childhood asthma prediction and has demonstrated better predictive performance in pediatric asthma prediction than existing conventional asthma prediction models, more research and external validation studies are still needed.

## 7. Directions for Future Research

The use of personalized medicine is expanding quickly, and it provides new methods for predictive modeling that can make a patient-centered approach easier. Genetic and epigenetic studies have provided a better understanding of the underlying pathological processes, and DNA methylation and microRNA expression are promising as potential biomarkers for predicting the trajectory of asthma; likewise, combinations of biomarkers may result in highly accurate asthma prediction. The use of models has facilitated the early diagnosis of asthma; however, clinical implementation is necessary to achieve a better result. On the other hand, asthma prediction models that have been developed using machine learning have made great progress in recent years, but external validation is needed.

## 8. Conclusions

Prediction models for childhood asthma have been proven to be functional in recognizing future asthmatics in high-risk groups of patients through their use in the preschool period, which is a crucial period for immune development and lung growth.

The parameters used in the different asthma prediction models have been generally validated in the populations that have been studied, favoring early diagnosis, intervention, and preventive measures.

Current knowledge of epigenetics, the use of biomarkers, and different types of algorithms in the prediction of asthma in children provide an opportunity to improve the accuracy of these diagnostic tools.

## Data Availability

Not applicable.

## Abbreviations

FEV_1_	Forced expiratory volume in one second.
FVC	Forced vital capacity.
EFL	Expiratory flow limitation.
ILC2	Innate lymphoid cells type 2.
AD	Atopic dermatitis.
TCRS	Tucson Children Respiratory Study.
FeNO	Fraction of exhaled nitric oxide.
VOC	Volatile organic compounds.
API	Asthma Predictive Index.
ML	Machine learning.
